# HIV-1 drug resistance and genetic diversity in a cohort of people with HIV-1 in Nigeria

**DOI:** 10.1097/QAD.0000000000003098

**Published:** 2021-10-07

**Authors:** Paul E. Oluniyi, Fehintola V. Ajogbasile, Shuntai Zhou, Iyanuoluwa Fred-Akintunwa, Christina S. Polyak, Julie A. Ake, Sodsai Tovanabutra, Michael Iroezindu, Morgane Rolland, Christian T. Happi

**Affiliations:** a Department of Biological Sciences, Faculty of Natural Sciences; b African Centre of Excellence for Genomics of Infectious Diseases (ACEGID), Redeemer's University, Ede, Osun State, Nigeria; c Lineberger Comprehensive Cancer Center, University of North Carolina at Chapel Hill, Chapel Hill, North Carolina; d U.S. Military HIV Research Program, Walter Reed Army Institute of Research; e Henry M. Jackson Foundation for the Advancement of Military Medicine, Silver Spring, Maryland, USA; f HJF Medical Research International, Abuja, Nigeria.

**Keywords:** dolutegravir, drug resistance, HIV-1, primer ID, subtype

## Abstract

This study was designed to provide information on the genetic diversity of HIV-1 and drug resistance mutations in Nigeria, as there is limited understanding of variants circulating in the country.

**Methods:**

We used an advanced next-generation sequencing platform, Primer ID, to: investigate the presence of high and low abundance drug resistance mutations; characterize preexisting Integrase Strand Transfer Inhibitor (INSTI) mutations in antiretroviral therapy (ART)-experienced but dolutegravir-naive individuals; detect recent HIV-1 infections and characterize subtype diversity from a cohort of people with HIV-1 (PWH).

**Results:**

HIV-1 subtype analysis revealed the predominance of CRF02_AG and subtype G in our study population. At detection sensitivity of 30% abundance, drug resistance mutations (DRMs) were identified in 3% of samples. At a sensitivity level of 10%, DRMs were identified in 27.3% of samples. We did not detect any major INSTI mutation associated with dolutegravir-resistance. Only one recent infection was detected in our study population.

**Conclusion:**

Our study suggests that dolutegravir-containing antiretroviral regimens will be effective in Nigeria. Our study also further emphasizes the high genetic diversity of HIV-1 in Nigeria and that CRF02_AG and subtype G are the dominant circulating forms of HIV-1 in Nigeria. These two circulating forms of the virus are largely driving the epidemic in the country.

## Introduction

The major defining characteristic of HIV-1 is its high genetic diversity, which is a result of a fast replication cycle, high mutation rate and high recombination rates. This high genetic diversity results in the presence of different variants of the virus in different regions of the world, and the emergence of new variants especially in areas with multiple circulating subtypes continues to occur [1,2].

There are four phylogenetic groups of HIV-1, these are the groups M (major), O (outlier), N (non-M/non-O), and the most recent group P [3–5]. The group M is majorly responsible for the global epidemic, includes more than 95% of globally sequenced HIV-1 samples and can be further classified into nine different subtypes (A–D, F–H, J, K), six A (A1–A6), and two F (F1 and F2) sub-subtypes together with 102 circulating recombinant forms (CRFs) reported (http://hiv-web.lanl.gov/CRFs/CRFs.html) and 100 unique recombinant forms (URFs) [1,6–8].

Subtype B is the predominant strain of HIV-1 in high-income countries whereas in low-income and middle-income countries (LMICs), especially in the African continent, the non-B subtypes together with several CRFs and URFs currently drive the epidemic. In sub-Saharan Africa, several studies have reported the presence of multiple HIV-1 subtypes along with a number of CRFs, such as CRF01_AE in Central Africa and CRF02_AG in West Africa [9–21].

The epidemic in Nigeria is complex. Several subtypes and other recombinants have been circulating, these include: subtype G, CRF06-cpx, CRF02-AG, sub-subtype A3, and other recombinants [14,18,20,22–26]. The high diversity of the virus within the country further contributes to the challenge of viral diagnosis, viral load determination, drug resistance testing, and HIV vaccine development. Although abundant information has been obtained from HIV type 1 (HIV-1) subtypes A, B, C, and CRF01_AE for HIV-1 vaccine design, Nigerian sequences are poorly represented [27]. As at 4 August 2021, there were only 84 complete HIV-1 genomes from Nigeria available on HIV-1 Los Alamos National Laboratory (LANL) Database (https://www.hiv.lanl.gov) compared with 978 from South Africa, 6679 from the United States of America, 372 from the United Kingdom or 1324 from Thailand.

Globally, the management of HIV infection has been seriously affected by the genetic diversity of the virus, because of the emergence of drug-resistant variants [28,29] and resultant treatment failure. A 2017 report from the WHO revealed that in quite a number of LMICs, about 10% of HIV-infected patients initiating antiretroviral therapy (ART) have preexisting HIV drug resistance to efavirenz and nevirapine [30,31]. These pretreatment drug resistance mutations can result in poor treatment outcomes and increased rate of death in adults and children. In response to the threat of drug resistance, many LMICs are moving away from nonnucleoside reverse transcriptase inhibitors (NNRTIs) and implementing policies to transition to dolutegravir as part of a more affordable and standardized ART. This is, however, not without its own challenges as most patients who have had access to dolutegravir reside in high-income countries and are infected with subtype B. Little is known about resistance pathways and mutation patterns of the virus to dolutegravir in PWH in Nigeria and Africa. Recent data have shown the possibility of dolutegravir resistance in places and countries where the predominant subtypes driving the epidemic are non-B subtypes, including Uganda [32], Cameroon [33], or South Africa [34]. There have also been reports suggesting that the mutational patterns of Integrase Strand Transfer Inhibitor (INSTI) (which dolutegravir is a class of) resistance might be different depending on the subtype [35–38].

In this study, we used an advanced next-generation sequencing platform, Primer ID, to determine the presence of high and low abundance drug resistance mutations, characterize preexisting INSTI mutations in ART-experienced but dolutegravir-naive individuals, detect recent HIV-1 infections and characterize subtype diversity from a cohort of PWH in Nigeria.

## Materials and methods

### Study design

Plasma samples were obtained from individuals who are part of the US Military HIV Research Program's African Cohort Study (AFRICOS) in Nigeria. AFRICOS is a prospective observational HIV-focused cohort, which seeks to longitudinally assess the impact of clinical practices, biological factors, and sociobehavioral issues on HIV infection and disease progression in five African countries [39]. Only adult participants were enrolled. Written informed consent was obtained from all participants. The study was approved by the institutional review boards of the Walter Reed Army Institute of Research, Redeemer's University, and the Nigerian Ministry of Defense. All samples were anonymized. The investigators have adhered to the policies for protection of human participants as prescribed in AR 70–25.

### Primer ID deep sequencing

Plasma samples were inactivated in buffer AVL and viral RNA was extracted according to the QiAmp viral RNA mini kit (Qiagen, Hilden Germany) manufacturer's instructions. Deep sequencing was carried out using the Primer ID (PID) Miseq Library Prep protocol previously described [40,41]. Full description of the PID sequencing protocol is available as Supplementary File 1.

### Near full-length genome sequencing

Full description of the methodology for the near full-length genome sequencing [42] is also available as Supplementary File 1.

### Sequence and phylogenetic analyses

Following sequencing, initial processing and construction of template consensus sequences (TCSs) was carried out using the Illumina bcl2fastq pipeline and the TCS pipeline version 1.33 (https://github.com/SwanstromLab/PID). This was followed by alignment of the TCSs to an HXB2 reference to remove sequences not mapped to the targeted region or with large deletions. Sequences from the *Protease (PR)*, *Reverse Transcriptase (RT)*, *Integrase (IN)*, and *V3* regions were aligned separately using MUSCLE version 3.8.31 [43] followed by calculation of raw (uncorrected) pairwise distances using the R ‘ape’ package [44]. Drug resistance was defined using a 2009 updated list of surveillance drug resistance mutations (https://hivdb.stanford.edu/pages/surveillance.html) to exclude polymorphisms that may not contribute to a resistance phenotype. We used a custom bash script to extract representative sequences from our template sequences for each sample. Subtype analysis was carried out using Stanford University HIVdb program version 9.0 (https://hivdb.stanford.edu/hivdb) and NCBI genotyping tool [45]. All nonproblematic complete genomes of HIV-1 from Nigeria were obtained from the HIV-1 Los Alamos National Laboratory (LANL) Database (80 in total) and codon-aligned with the eight near full-length genomes (NFLGs) obtained from this study in order to understand the evolutionary relationship between the near full-length genomes obtained from this study and previous genomes from Nigeria. Codon alignment was performed using Gene Cutter (https://www.hiv.lanl.gov/content/sequence/GENE_CUTTER/cutter.html). A maximum likelihood phylogenetic tree was constructed using IQTREE [46] with the GTR+F+R7 model.

## Results

### Samples’ summary

Using the PID protocol, sequences were obtained from samples from 42 PWH enrolled in the AFRICOS in Lagos (*n* = 38) and Abuja (*n* = 4). Samples were collected between 2016 and 2018 and viral loads ranged from 1250 to 1 310 000 copies/ml. Thirty-three *IN* sequences (78.6%), 33 *PR* sequences (78.6%), 17 *RT* sequences (40.5%), 24 *V1V3* sequences (57.1%), and 8 P17 sequences (19%) were obtained from sequenced samples (Supplementary Table 1).

Additionally, near full-length genomes, here referred to as genomes, were obtained from plasma samples collected from eight participants between 2014 and 2015 with viral load ranging between 83 700 and 1 660 000 copies/ml. One genome per participant was obtained.

### Subtype identification

**Fig. 1:**
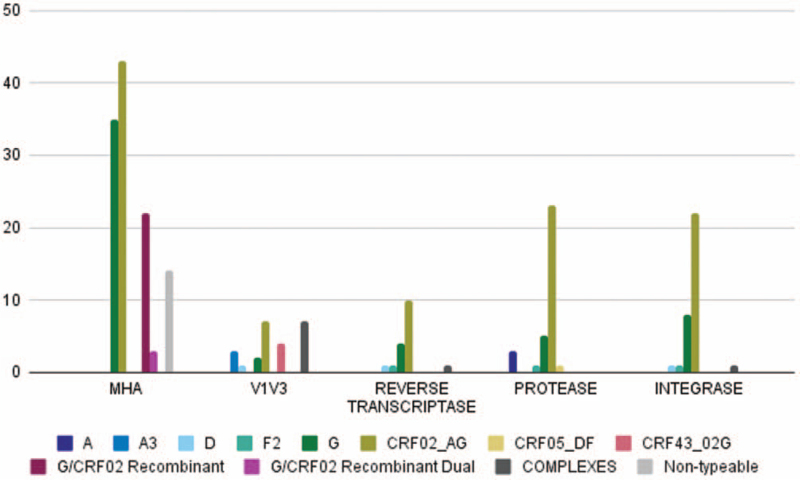
Results of *integrase (IN)*, *protease (PR)*, *reverse transcriptase (RT)*, *V1V3*, and MHA subtype analysis.

Subtype analysis was carried out for four of the five gene fragments from which we obtained the most sequences among the sequenced samples and this revealed that of the 33 *integrase* sequences obtained, 22 (66.7%) were CRF02_AG, 8 (24.2%) were subtype G, 1 (3%) was CRF06_CPX, 1 (3%) was sub-subtype F2, and 1 (3%) was subtype D. Subtype analysis of the 33 *protease* sequences revealed that 23 (69.7%) were CRF02_AG, 5 (15.2) were subtype G, 3 (9.1%) were subtype A, 1 (3%) was CRF05_DF, and 1 (3%) was sub-subtype F2. Of the 17 *reverse transcriptase* sequences obtained, 10 (58.8%) were CRF02_AG, 4 (23.5%) were subtype G, 1 (5.9%) was CRF06_CPX, 1 (5.9%) was sub-subtype F2, and 1 (5.9%) was subtype D. Of the 24 *V1V3* sequences obtained, 7 (29.2%) were CRF02_AG, 7 (29.2%) were complexes (37_CPX, 09_CPX, 45_CPX, 11_CPX and 56_CPX), 4 (16.7%) were CRF43_02G, 3 (12.5%) were subtype A3, 2 (8.3%) were subtype G, and 1 (4.2) was subtype D. These results are in line with data obtained by testing 189 samples from the same cohort with a multiregion hybridization assay (MHA) [47] (Fig. 1) and also data obtained by screening for intersubtype recombinants (Supplementary File 2).

Nine (21.4%) of the 42 sequenced samples had sequences for the four genes used for subtyping (*integrase, protease, reverse transcriptase* and *V1V3*), 18 (42.9%) had sequences for three of the four genes, nine (21.4%) had sequences for two of the four genes whereas 2 (4.8%) had sequences for one of the four genes.

**Fig. 2:**
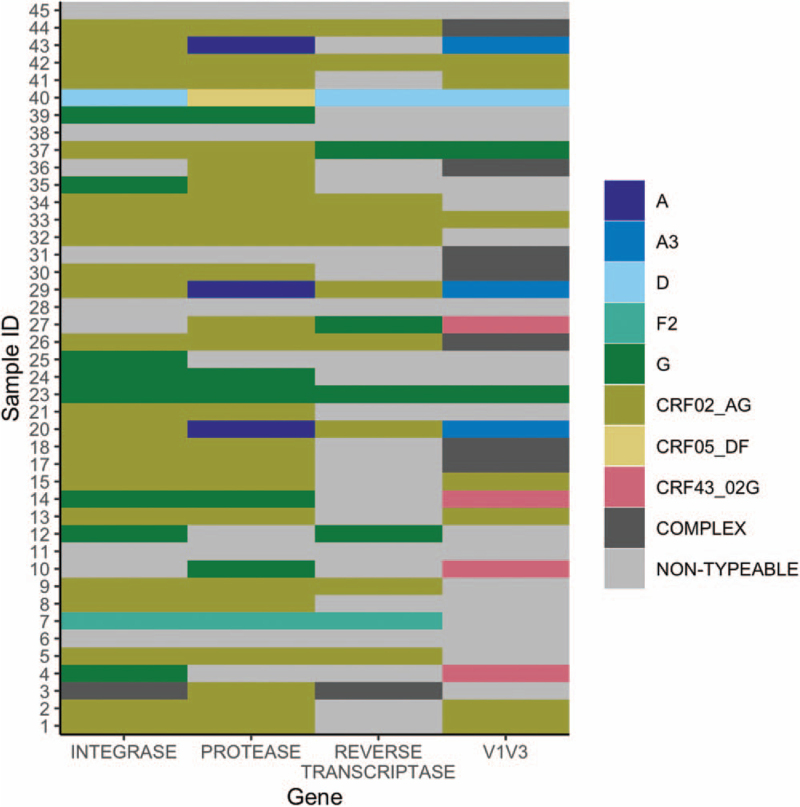
Subtype diversity across different genes (*IN*, *PR*, *RT*, and *V1V3*) from sequenced samples.

Further analysis showed that three (33.3%) of the nine isolates sequenced in the four fragments had subtype concordance in all four fragments while six (66.7%) showed discordant subtypes. Of the 18 samples that had sequences for three genes, nine (50%) had subtype concordance in the three genes whereas nine (50%) showed discordant subtypes while of the nine isolates sequenced in two of the four genes, five (55.6%) had subtype concordance in the two genes while four (44.4%) had subtype discordance (Fig. 2).

**Fig. 3:**
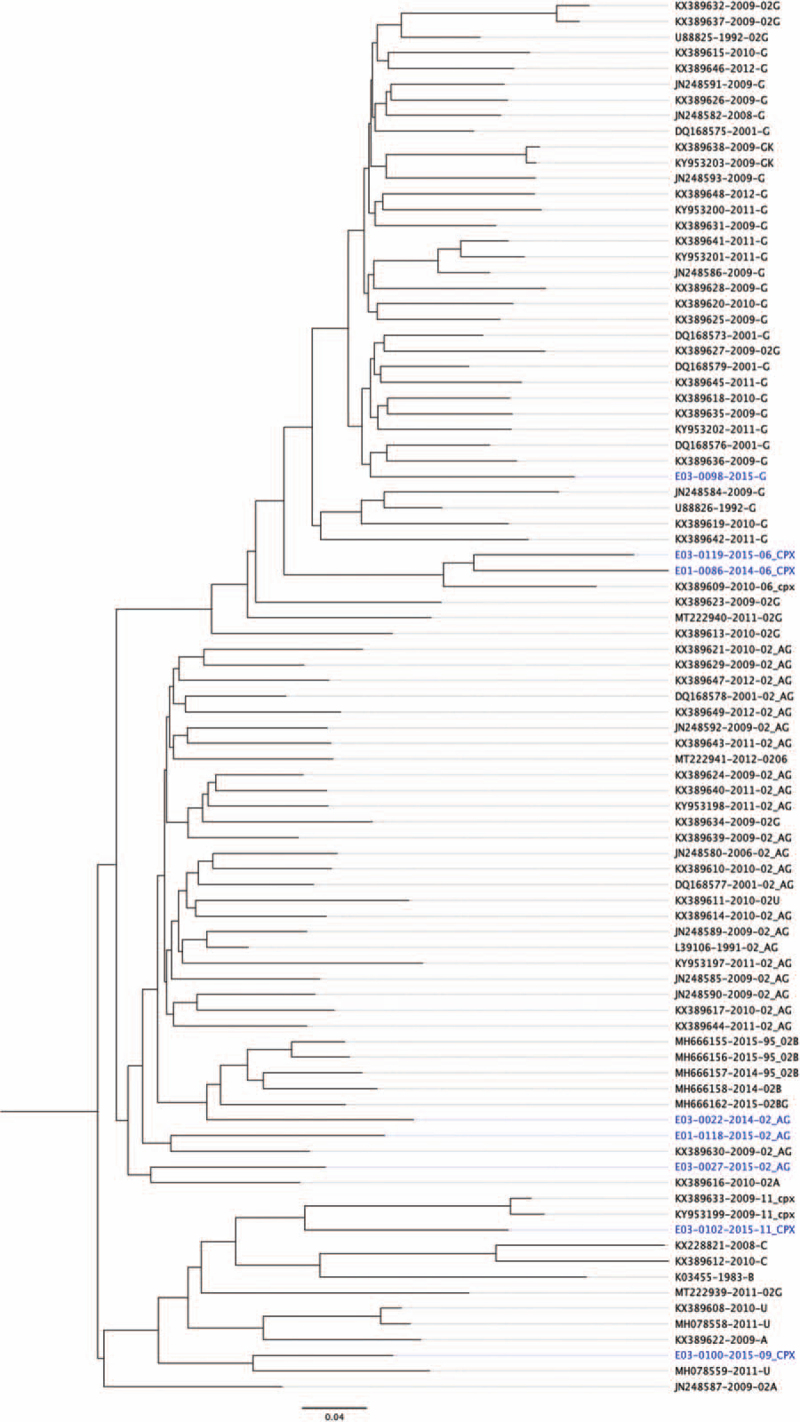
Mid-point rooted maximum likelihood phylogenetic tree showing relationship between the eight near full-length genome sequences obtained from this study (coloured blue) and all full nonproblematic Nigerian HIV-1 genomes (80 in total) available in the HIV-1 Los Alamos National Laboratory (LANL) database.

Subtype and phylogenetic analysis was also carried out for the eight HIV-1 genomes obtained from this study (Fig. 3). This revealed that of the eight genomes, three (37.5%) were CRF02_AG, two (25%) were CRF06_CPX, one (12.5%) was subtype G, one (12.5%) was CRF09_CPX, and one (12.5%) was CRF11_CPX.

### Time of infection

Using parameters previously described [48], we estimated recent HIV-1 infection based on the sequence diversity (*π*) and the first quintile of the pairwise comparison.

Both *RT* and *V1V3* sequences were used for recency analysis. Specimens were classified as recent HIV infection (within 9 months of transmission), chronic infection, or indeterminate. Only one recent infection was observed in this study.

### Identification of drug resistance mutations

#### Gene fragments drug resistance mutation analysis

The sensitivity for the detection of drug resistance mutations (DRMs) that are not fixed (i.e. at 100%) is defined by the viral sequence population's sampling depth. Grouping specimens based on the sequence sampling depth, and thus the levels of sensitivity of minor variants, is therefore, essential [49]. The PID sequencing allows us to tag each sequenced viral RNA with a unique molecular ID (UMI) and the TCS number of one specimen represents the sampling depths. Using this approach, we could greatly reduce the sequencing error and calculate the true abundance of mutations with confidence intervals. To compare the mutations at different abundance levels in the study population, we grouped the participants based on the minimal number of TCS at each region based on the detection sensitivity. The detection sensitivity is defined as the minimal percentage of mutations we can detect with 95% confidence given a certain number of TCS (sampling depth) using binomial distribution. We grouped our samples based on TCS counts of at least 10 or 34 (Table 1) and this allowed us to detect mutations at sampling depths of 30 and 10% abundance, respectively with 95% confidence of detection.

**Table 1:** Number of samples with template consensus sequence of at least 10 (sampling depth of 30%) or 34 (sampling depth of 10%).

Sampling depth (%)	PR	RT	IN
30	9	10	7
10	9	2	20

IN, integrase; PR, protease; RT, reverse transcriptase.

At detection sensitivity of 30% abundance, DRMs were identified in 3% of the samples (1 of 33) in *IN*, in 3% (1 of 33) in *PR*, and in 17.6% (3 of 17) in the *RT* region. At a sensitivity level of 10%, DRMs were identified in 27.3% of samples (9 of 33) in *IN*, 6.1% (2 of 33) in *PR*, and in 5.9% (1 of 17) in the *RT* region.

Q148R was the only *IN* mutation observed in one sample at 30% sensitivity level while at 10% sensitivity level, the *IN* mutations detected included: L74M (in four samples), T97A (in four samples), F121Y (in one sample), T66A (in two samples), S147G (in one sample), Y143C (in one sample), and Y143H (in two samples).

*PR* mutations observed at 30% sensitivity level included: F53L (in one sample) while at 10% sensitivity level, the following mutations were detected: M46I (in two samples), F53L (in one sample), I54T (in one sample), and I54S (in one sample).

*RT* mutations observed at 30% sensitivity level included: P225H (in two samples), L74I (in one sample), M184I (in one sample), K103N (in two samples) while at 10% sensitivity level, each of the mutations L100I, K101E, K103N, V106A, V106 M, Y181C, Y188H, G190A and M230L were observed in one sample.

A total of 14.3% (6 of 42) of individuals in this study had drug resistance mutations in at least two genes while none had mutations in all three drug resistance genes (*IN, PR*, and *RT*). Also, out of 10 individuals in which we observed *IN* mutations, eight (80%) had CRF02_AG infections while one was a subtype G and one a subtype D infection; of three individuals in which we observed *PR* mutations, two had CRF02_AG infections and one was a subtype G infection; of four individuals in which we observed *RT* mutations, two had CRF02_AG infections, one was a subtype D and one was a CRF06_CPX infection.

### Near full-length genomes drug resistance mutation analysis

Drug resistance analysis carried out on the eight genome sequences obtained from this study revealed the presence of *IN* mutations E157Q (in one sample) and Q146QR (in one sample). Nonnucleoside reverse transcriptase mutations (NNRTIs) V106I (in one sample) and V179E (in two samples) were also observed.

The *IN* mutations observed in this study occurred in CRF06_CPX and CRF02_AG sequences while the NNRTIs occurred in CRF09_CPX, G and CRF06_CPX sequences.

## Discussion

This study shows the circulation of HIV-1 subtypes/sub-subtypes A, A3, G, CRF02_AG, D, F2, CRF37_CPX, CRF06_CPX, CRF09_CPX, CRF45_CPX, CRF11_CPX, CRF56_CPX, and CRF05_DF in different proportions among PWH in Nigeria. The predominance of CRF02_AG (22 out of 33 in *integrase*, 23 out of 33 in *protease*, 10 out of 17 in *reverse transcriptase*, seven out of 24 in *V1V3*, three out of eight in whole genome sequences) and subtype G (8 out of 33 in *integrase*, five out of 33 in *protease*, four out of 17 in *reverse transcriptase*, two out of 24 in *V1V3*, and one out of eight in whole genome sequences) is in line with previous studies carried out in Nigeria [14,18,20–21,25,50–56]. CRF02_AG and subtype G are the dominant circulating forms of HIV-1 in Nigeria and they are largely driving the epidemic in the country. The observed dominant spread of CRF02_AG in West Africa may be attributed to the replicative fitness it confers over subtypes A and G in the same geographical region [57,58]. The observation of a wide array of subtypes among a small number of individuals in this study further emphasizes the high genetic diversity of the virus in Nigeria. Previous studies have shown that movement of people from one country to another has contributed largely to the spread of HIV-1 diversity worldwide [20]. In developing countries, the migration of rural populations because of poverty, famine, civil wars, and so forth have been additional contributing factors to the genetic diversity of the virus [59,60].

This study also revealed recombination among the isolates from which we obtained sequences from more than one genomic region that was genotyped. Six (66.7%) of the nine samples sequenced in four genomic regions used for subtyping (*integrase*, *protease*, *reverse transcriptase* and *V1V3*) had disparate subtypes. For samples in which three genomic regions were sequenced, nine (50%) of 18 samples had different subtypes while four (44.4%) of nine samples in which two genomic regions were sequenced had discordant subtypes. This could be as a result of marked genetic heterogeneity of the virus in these gene regions and demonstrates the complex diversity of the HIV-1 strains circulating in Nigeria. This also emphasizes the need to sequence the complete genomes of the virus in Nigeria to better understand subtype diversity in the country; prior to our study, there have only been 84 whole genome sequences of HIV-1 from Nigeria deposited in public databases, here we added eight new sequences. This can in turn help improve drug and vaccine development processes. Previous studies have noted that in regions where multiple subtypes are in circulation, there is an increased possibility of recombinant strains arising [61]. Recombinant strains of HIV-1 usually have biological advantages over their parental strains, and some of these advantages could manifest in different forms, some of which include: improved viral fitness, enhanced co-receptor usage, and so forth. This has a lot of implications for clinical management of patients in the region. It can affect the sensitivity of diagnostic kits and can result in reduction in the susceptibility of patients to antiretroviral drugs [20,62,63].

A major objective of this study was to understand if there were preexisting INSTI mutations in ART-experienced but dolutegravir-naive individuals that confer resistance to dolutegravir present in our study population. The WHO and the US President's Emergency Plan for AIDS Relief (PEPFAR) are recommending regimens that include tenofovir (as the disoproxil fumarate or alafenamide formulation), together with lamivudine and dolutegravir, as a first-line regimen to replace efavirenz-based regimens in LMICs and this is now being rolled out in Nigeria (starting from 2018). The high genetic barrier to dolutegravir resistance when administered as a dolutegravir (DTG)-containing combination antiretroviral therapy (cART) has been demonstrated *in vivo* but in majorly HIV-1 subtype B-infected individuals, so little/nothing is known about the genetic correlates of resistance for nonsubtype B infections [31]. At 30% sampling depth, we detected the INSTI mutation Q148R while at 10% sampling depth, we detected INSTI mutations L74M, T97A, F121Y, T66A, S147G, Y143C/H in varying numbers of samples. Q148R, which is a major INSTI mutation was the only INSTI mutation observed at 30% sensitivity level, it is a nonpolymorphic mutation selected in patients receiving raltegravir (RAL) and elvitegravir (EVG) but has been reported in patients with virologic failure during dolutegravir monotherapy or salvage therapy [64,65]. However, these studies were restricted to subtype B.

Other major INSTI mutations observed in our study at 10% sensitivity level included: T66A, S147G, Y143C/H. T66A is a nonpolymorphic mutation selected in patients receiving EVG and RAL. It, however, has minimal effect on DTG susceptibility [66–68]. S147G is a nonpolymorphic mutation selected in patients receiving EVG, it reduces EVG susceptibility by five-fold but has been shown to have minimal if any effect on DTG susceptibility [66,69]. Y143C/H are also nonpolymorphic mutations selected by RAL but do not reduce DTG susceptibility [70,71]. Accessory INSTI resistance mutations observed in our study occurred only at a sensitivity level of 10% and they include L74M, T97A, F121Y. L74M, and T97A were the most common accessory mutations that we observed, which is in line with previous studies carried out in sub-Saharan Africa [72]. Also, similar to previous reports, L74M and T97A were more frequent in participants infected with subtype A, G, and recombinant viruses [73]. L74I/M are polymorphic mutations, which are commonly selected by all three INSTI drugs, they occur at varying degrees in antiretroviral-naive populations (usually between 0.5 and 20%) and they are highly prevalent in subtypes, A, G, and A/G recombinants. However, unless they occur in combination with other major INSTI mutations, mainly Q148H/K/R, they do not affect INSTI susceptibility [74]. In the same line, T97A, on its own, has little/no effect on susceptibility to INSTIs but when occurring in combination with Y143 and N155H major resistance mutations, it can cause significant reduction in susceptibility to RAL and EVG [68,75]. F121Y is a nonpolymorphic mutation that has been shown to have little to no effect on DTG susceptibility [76,77]. Our findings from this study suggest that dolutegravir-containing antiretroviral regimens will be effective in Nigeria. This is important as Nigeria begins to adapt and implement the recent guidelines advocating for dolutegravir-based first-line treatment. It is, however, important to monitor patients as they begin on dolutegravir in order to obtain first hand data on response to treatment and resistance mutations selected by dolutegravir in non-B subtypes. It is also important to understand if resistance pathways in non-B subtypes are different as previous studies have postulated a different resistance pathway for dolutegravir that involves the G118R mutation selected mainly in nonsubtype B viruses [78].

Using the Primer ID approach also allowed us to find a class of mutations notably F53L protease mutation and K103N reverse transcriptase mutation, which were present in a similar number of samples irrespective of genome sampling depth. This implies that whenever these mutations are present in a virus population, they are usually high in number and relatively stable over time [79]. We also only detected one recent infection in our study, which implies that almost all samples were collected at the late stage of infection.

In conclusion, we did not detect any major INSTI mutation associated with dolutegravir resistance, and therefore, suggest that dolutegravir-containing antiretroviral regimens will be effective in Nigeria.

Our study further emphasizes the high genetic diversity of HIV-1 in Nigeria and that CRF02_AG and subtype G are the dominant circulating forms of HIV-1 in Nigeria. These two circulating forms of the virus are largely driving the epidemic in the country.

## Acknowledgements

We thank Miss Jessica Uwanibe, Dr Adeyemi Kayode, Dr Trevor Crowell, and Dr Bethany Dearlove for helpful scientific discussions/manuscript review, for providing assistance with laboratory experiments and helping with figures creation/editing.

We thank Meera Bose, Daniel Silas, Sandra Mendoza Guerrero, Marty Nau, Kultida Poltavee, and Leigh Anne Eller for technical assistance. We thank Drs Nelson Michael and Merlin Robb for initiating this project.

This work is made possible by support from Flu Lab and a cohort of generous donors through TED's Audacious Project, including the ELMA Foundation, MacKenzie Scott, the Skoll Foundation, and Open Philanthropy. This work was also supported by grants from the National Institute of Allergy and Infectious Diseases (https://www.niaid.nih.gov), NIH-H3Africa (https://h3africa.org) (U01HG007480 and U54HG007480 to C.T.H.), the World Bank grant (worldbank.org) (ACE IMPACT project to C.T.H.), the President's Emergency Plan for AIDS Relief via a cooperative agreement between the Henry M. Jackson Foundation for the Advancement of Military Medicine, Inc., and the U.S. Department of Defense (W81XWH-18–2–0040 to J.A.A.). The views expressed are those of the authors and should not be construed to represent the positions of the US Army, the Department of Defense, or the Department of Health and Human Services.

Funding: This work is made possible by support from Flu Lab and a cohort of generous donors through TED's Audacious Project, including the ELMA Foundation, MacKenzie Scott, the Skoll Foundation, and Open Philanthropy. This work was also supported by grants from the National Institute of Allergy and Infectious Diseases (https://www.niaid.nih.gov), NIH-H3Africa (https://h3africa.org) (U01HG007480 and U54HG007480 to C.T.H.), the World Bank grant (worldbank.org) (ACE IMPACT project to C.T.H.), the President's Emergency Plan for AIDS Relief via a cooperative agreement between the Henry M. Jackson Foundation for the Advancement of Military Medicine, Inc., and the US Department of Defense (W81XWH-18-2-0040 to J.A.A.).

Author contributions: P.E.O. and F.V.A. wrote manuscript draft, P.E.O., F.V.A., I.F., and S.T. carried out laboratory experiments, P.E.O. and S.Z. carried out data analysis, S.Z. reviewed the manuscript, J.A.A., C.P., and M.I. conducted the AFRICOS study, M.R. oversaw data analysis, M.R. and C.T.H. designed the study, oversaw the project, and reviewed the manuscript.

Accession numbers: all raw sequencing data obtained from this study have been submitted to NCBI SRA (BioProject accession number: PRJNA761121). Template consensus sequences (TCSs) and individual representative sequences used for subtyping are available on GitHub (https://github.com/pauloluniyi/HIV-1_Paper).

Abbreviations: a full list of abbreviations is available as Supplementary File 3.

### Conflicts of interest

There are no conflicts of interest.

## Supplementary Material

Supplemental Digital Content

## Supplementary Material

Supplemental Digital Content

## Supplementary Material

Supplemental Digital Content

## Supplementary Material

Supplemental Digital Content
