# Supplementary material for: HIV-1 drug resistance and genetic diversity in a cohort of people with HIV-1 in Nigeria
Source: AIDS. 2021 Oct 7;36(1):137–46. doi: 10.1097/QAD.0000000000003098 (PMC8654252; doi:10.1097/QAD.0000000000003098)
Supplement: Supplemental Digital Content [file aids-36-137-s001.docx]

**Supplementary File 1**

**Primer ID Deep Sequencing**

Complementary DNA (cDNA) was synthesized from the extracted viral RNA using a cDNA primer mixture targeting the protease (PR), reverse transcriptase (RT), integrase (IN) and *env* V3 coding regions with a block of random nucleotides in each cDNA primer serving as the PID, and SuperScript III RT. Two rounds of Polymerase chain reaction (PCR) were used to further amplify the synthesized cDNA and incorporate Illumina indexed adapters with KAPA2G Robust and KAPA HiFi PCR kits, respectively. The libraries (2nd round PCR products) were resolved on 1.2% agarose gel for confirmation. Expected fragment sizes (850bp and 600bp) were excised and purified using the Qiagen QIAquick gel extraction kit according to manufacturer’s instructions. Gel-purified libraries were quantified using Applied Biosystems Qubit DsDNA Assay Kit. Normalized libraries were pooled and sequenced on the Illumina MiSeq Platform with 300 base paired-end reads.

**Near Full-Length Genome Sequencing**

Viral RNA was extracted from plasma samples using a QIAamp viral RNA kit (Qiagen). Complementary DNA was synthesized with SuperScript III Reverse Transcriptase (Invitrogen) using Oligo (dT) primer for near full length genomes (HXB2:789–9496) or 3’-half genomes (HXB2: 4559–9496) amplification and a specific primer (JL68RV2: 5’- CTTCTTCCTGCCATAGGAGATGCCTAAG-3’) for 5’-half genome amplification (HXB2: 789–5852). Single genome amplification was done via nested PCR using the Advantage GC Genomic LA kit (Clontech Laboratories, Inc., USA) as previously described [42]. Primers used for genome amplification are listed below:

**Full-length (FL) First round primers:**

Msf12b 5’-AAATCTCTAGCAGTGGCGCCCGAACAG-3’

Uninef-7’ 5’-GCACTCAAGGCAAGCTTTATTGAGGCTT-3’

**FL second round primers:**

Gag763 5’-TGACTAGCGGAGGCTAGAAGGAGAGA-3’

Tatanef 5’- GCAGCTGCTTATATGCAGGATCTGAGGG -3

**Left-Half (LH) First round primers:**

Msf12b 5’-AAATCTCTAGCAGTGGCGCCCGAACAG-3’

JL68Rv2 5’- CTTCTTCCTGCCATAGGAGATGCCTAAG-3’

**LH second round primers:**

Gag763 5’-TGACTAGCGGAGGCTAGAAGGAGAGA-3’

TatAD’ 5’- TTCCCGGRTGKTTCCAGGGCTCTA-3’

**Right-Half (RH) First round primers:**

PolJv2 5’-GAAGCYATGCATGGACAAGTRGA-3’

Uninef-7’ 5’-GCACTCAAGGCAAGCTTTATTGAGGCTT-3’

**RH second round primers:**

PolK3 5’- TAAARYTAGCAGGAAGATGGCCAGT-3’

Tatanef 5’- GCAGCTGCTTATATGCAGGATCTGAGGG -3’

PCR products were purified and sequenced using an Applied Biosystems 3730 DNA Analyzer.
