# Supplementary material for: HIV-1 drug resistance and genetic diversity in a cohort of people with HIV-1 in Nigeria
Source: AIDS. 2021 Oct 7;36(1):137–46. doi: 10.1097/QAD.0000000000003098 (PMC8654252; doi:10.1097/QAD.0000000000003098)
Supplement: Supplemental Digital Content [file aids-36-137-s002.docx]

**Supplementary Table 1:** Gene sequences obtained from sequenced samples

| **S/N** | **Sample ID** | **IN** | **PR** | **RT** | **V1V3** | **P17** |
| --- | --- | --- | --- | --- | --- | --- |
| 1. | RV1 | 1 | 1 | 0 | 1 | 0 |
| 2. | RV2 | 1 | 1 | 0 | 1 | 1 |
| 3. | RV3 | 1 | 1 | 1 | 0 | 1 |
| 4. | RV4 | 1 | 0 | 0 | 1 | 0 |
| 5. | RV5 | 1 | 1 | 1 | 1 | 0 |
| 6. | RV6 | 1 | 1 | 0 | 0 | 0 |
| 7. | RV7 | 1 | 1 | 1 | 0 | 0 |
| 8. | RV8 | 1 | 1 | 0 | 1 | 1 |
| 9. | RV9 | 1 | 1 | 1 | 1 | 1 |
| 10. | RV10 | 1 | 1 | 0 | 1 | 0 |
| 11. | RV11 | 1 | 1 | 0 | 0 | 0 |
| 12. | RV12 | 1 | 1 | 1 | 0 | 0 |
| 13. | RV13 | 1 | 1 | 0 | 1 | 1 |
| 14. | RV14 | 1 | 1 | 0 | 1 | 0 |
| 15. | RV15 | 1 | 1 | 0 | 1 | 1 |
| 16. | RV17 | 1 | 1 | 0 | 1 | 1 |
| 17. | RV18 | 1 | 1 | 0 | 1 | 1 |
| 18. | RV20 | 1 | 1 | 1 | 1 | 1 |
| 19. | RV21 | 1 | 1 | 0 | 0 | 1 |
| 20. | RV23 | 1 | 1 | 1 | 1 | 0 |
| 21. | RV24 | 1 | 0 | 0 | 0 | 0 |
| 22. | RV25 | 1 | 0 | 0 | 0 | 0 |
| 23. | RV26 | 1 | 1 | 1 | 1 | 0 |
| 24. | RV27 | 0 | 1 | 1 | 1 | 0 |
| 25. | RV28 | 0 | 0 | 0 | 0 | 0 |
| 26. | RV29 | 1 | 1 | 1 | 1 | 0 |
| 27. | RV30 | 1 | 1 | 0 | 1 | 0 |
| 28. | RV31 | 0 | 0 | 0 | 1 | 0 |
| 29. | RV32 | 1 | 1 | 1 | 0 | 0 |
| 30. | RV33 | 1 | 1 | 1 | 1 | 0 |
| 31. | RV34 | 1 | 1 | 1 | 0 | 0 |
| 32. | RV35 | 1 | 1 | 0 | 0 | 0 |
| 33. | RV36 | 0 | 1 | 0 | 1 | 0 |
| 34. | RV37 | 1 | 1 | 1 | 1 | 0 |
| 35. | RV38 | 0 | 0 | 0 | 0 | 0 |
| 36. | RV39 | 1 | 1 | 0 | 0 | 0 |
| 37. | RV40 | 1 | 1 | 1 | 1 | 0 |
| 38. | RV41 | 1 | 1 | 0 | 1 | 0 |
| 39. | RV42 | 1 | 1 | 1 | 1 | 0 |
| 40. | RV43 | 1 | 1 | 0 | 1 | 0 |
| 41. | RV44 | 1 | 1 | 1 | 1 | 0 |
| 42. | RV45 | 0 | 0 | 0 | 0 | 0 |

**Note: 0** - Sequencing failure, **1** - Sequencing success
