# Supplementary material for: HIV-1 drug resistance and genetic diversity in a cohort of people with HIV-1 in Nigeria
Source: AIDS. 2021 Oct 7;36(1):137–46. doi: 10.1097/QAD.0000000000003098 (PMC8654252; doi:10.1097/QAD.0000000000003098)
Supplement: Supplemental Digital Content [file aids-36-137-s003.docx]

**Supplementary File 2**

**Inter-subtype Recombination Analysis**

The gene sequences (Table 1) and near full-length genomes (Table 2) in this study were screened for the evidence of inter-subtype recombination by the Recombination Identification Program (<https://www.hiv.lanl.gov/content/sequence/RIP/RIP.html>).  This revealed that the majority of the individuals in this study were infected by inter-subtype recombinants and is in line with the results of our subtyping analysis.

**Table 1: Gene Sequences**

| **S/N** | **Sample ID** | **IN** | **PR** | **RT** | **V1V3** |
| --- | --- | --- | --- | --- | --- |
| 1. | RV1 | CRF02_AG | CRF02_AG | - | CRF02_AG |
| 2. | RV2 | CRF02_AG | CRF02_AG | - | CRF02_AG |
| 3. | RV3 | CRF06_CPX | CRF02_AG | CRF06_CPX | - |
| 4. | RV4 | G | - | - | CRF43_02G |
| 5. | RV5 | CRF02_AG | CRF02_AG | CRF02_AG | - |
| 6. | RV6 | - | - | - | - |
| 7. | RV7 | F2 | F2 | F2 | - |
| 8. | RV8 | CRF02_AG | CRF02_AG | - | - |
| 9. | RV9 | CRF02_AG | CRF02_AG | CRF02_AG | - |
| 10. | RV10 | - | G | - | CRF43_02G |
| 11. | RV11 | - | - | - | - |
| 12. | RV12 | G | - | G | - |
| 13. | RV13 | CRF02_AG | CRF02_AG | - | CRF02_AG |
| 14. | RV14 | G | G | - | CRF43_02G |
| 15. | RV15 | CRF02_AG | CRF02_AG | - | CRF02_AG |
| 16. | RV17 | CRF02_AG | CRF02_AG | - | CRF37_CPX |
| 17. | RV18 | CRF02_AG | CRF02_AG | - | CRF37_CPX |
| 18. | RV20 | CRF02_AG | A | CRF02_AG | A3 |
| 19. | RV21 | CRF02_AG | CRF02_AG | - | - |
| 20. | RV23 | G | G | G | G |
| 21. | RV24 | G | G | - | - |
| 22. | RV25 | G | - | - | - |
| 23. | RV26 | CRF02_AG | CRF02_AG | CRF02_AG | A1/CRF19_CPX |
| 24. | RV27 | - | CRF02_AG | G | CRF43_02G |
| 25. | RV28 | - | - | - | - |
| 26. | RV29 | CRF02_AG | A | CRF02_AG | A3 |
| 27. | RV30 | CRF02_AG | CRF02_AG | - | CRF45_CPX |
| 28. | RV31 | - | - | - | CRF56_CPX |
| 29. | RV32 | CRF02_AG | CRF02_AG | CRF02_AG | - |
| 30. | RV33 | CRF02_AG | CRF02_AG | CRF02_AG | CRF02_AG |
| 31. | RV34 | CRF02_AG | CRF02_AG | CRF02_AG | - |
| 32. | RV35 | G | CRF02_AG | - | - |
| 33. | RV36 | - | CRF02_AG | - | CRF56_CPX |
| 34. | RV37 | CRF02_AG | CRF02_AG | G | G |
| 35. | RV38 | - | - | - | - |
| 36. | RV39 | G | G | - | - |
| 37. | RV40 | D | CRF05_DF | D | D |
| 38. | RV41 | CRF02_AG | CRF02_AG | - | CRF02_AG |
| 39. | RV42 | CRF02_AG | CRF02_AG | CRF02_AG | CRF02_AG |
| 40. | RV43 | CRF02_AG | A | - | A3 |
| 41. | RV44 | CRF02_AG | CRF02_AG | CRF02_AG | CRF56_CPX |
| 42. | RV45 | - | - | - | - |

**Note: ‘-’ = Sequencing failure**

**Table 2:**  Near full-length genomes

| **S/N** | **Sample ID** | **Recombinants** |
| --- | --- | --- |
| 1. | E03-0119 | CRF06_CPX |
| 2. | E01-0118 | CRF02_AG |
| 3. | E03-0100 | CRF09_CPX |
| 4. | E03-0098 | G |
| 5. | E03-0022 | CRF02_AG |
| 6. | E01-0086 | CRF06_CPX |
| 7. | E03-0027 | CRF02_AG |
| 8. | E03-0102 | CRF11_CPX |
