# Supplementary material for: HIV-1 drug resistance and genetic diversity in a cohort of people with HIV-1 in Nigeria
Source: AIDS. 2021 Oct 7;36(1):137–46. doi: 10.1097/QAD.0000000000003098 (PMC8654252; doi:10.1097/QAD.0000000000003098)
Supplement: Supplemental Digital Content [file aids-36-137-s004.docx]

**Supplementary File 3**

**Abbreviations**

**AFRICOS -** African Cohort Study

**ART -** Antiretroviral Therapy

**CRF -** Circulating Recombinant Form

**DTG -** Dolutegravir

**EVG -** Elvitegravir

**URF -** Unique Recombinant Form

**DRM -** Drug Resistance Mutation

**INSTI -** Integrase Strand Transfer Inhibitor

**NFLG -** Near Full-Length Genome

**NNRTI -** Non-nucleoside Reverse Transcriptase Inhibitor

**PLWH -** People Living with HIV-1

**RAL -** Raltegravir

**TCS -** Template Consensus Sequence
